# 重型/危重型新型冠状病毒肺炎（COVID-19）患者凝血功能障碍与细胞因子风暴综合征关系的研究进展

**DOI:** 10.3760/cma.j.issn.0253-2727.2021.08.019

**Published:** 2021-08

**Authors:** 婕 张, 晓玲 高, 林花 杨

**Affiliations:** 1 山西医科大学，太原 030001 Shanxi Medical University, Taiyuan 030001; 2 山西医科大学第二医院呼吸与危重症医学科，太原 030001 Department of Respiratory and Critical Care Medicine, The Second Hospital of Shanxi Medical University, Taiyuan 030001, China; 3 山西医科大学第二医院血液科，太原 030001 Department of Hematology, The Second Hospital of Shanxi Medical University, Taiyuan 030001, China

血管内高凝状态及微血栓形成是新型冠状病毒肺炎（COVID-19）患者发展为重症的关键节点[1]。目前认为，COVID-19凝血功能障碍是一个动态变化的过程，其机制与严重急性呼吸综合征冠状病毒-2（SARS-CoV-2）感染后免疫系统逐步失衡所致细胞因子风暴综合征（CSS）介导的广泛血管内高凝相关，但具体机制尚未完全阐明[2]–[3]。本文对重型/危重型COVID-19凝血功能障碍及其与CSS相互作用关系的最新研究进展做一综述，为这一疾病研究及临床诊治提供参考。

一、重型/危重型COVID-19凝血功能指标的改变

COVID-19可影响几乎所有的实验室凝血功能指标，但这些指标的改变程度及其与疾病严重程度和死亡率的相关性各不相同。

1. D-二聚体：D-二聚体是一种纤维蛋白降解产物，在血栓事件中其水平显著升高，提示凝血过度激活和纤维蛋白溶解亢进。36％～46％的COVID-19患者D-二聚体升高[4]。研究发现，入院时D-二聚体升高与COVID-19疾病严重程度及死亡风险增加密切相关[5]。当D-二聚体>0.635 mg/L时有助于诊断重型患者，当D-二聚体>1.5 mg/L可有效预测静脉血栓栓塞症（VTE），当D-二聚体≥2.0 mg/L可有效预测COVID-19患者的住院死亡[6]–[8]。目前越来越多的证据支持，COVID-19患者D-二聚体水平升高不仅可以预测血栓事件，还可以作为CSS、弥散性血管内凝血（DIC）、急性呼吸窘迫综合征（ARDS）的风险分层及进展评估工具，为区分疾病严重程度、预测死亡及预后提供有效帮助[9]–[12]。

2. 纤维蛋白原及纤维蛋白原降解产物（FDP）：纤维蛋白原是血栓形成的重要反应底物，其水平升高提示机体处于血栓形成前的高凝状态。FDP是纤维蛋白原或纤维蛋白被分解后生成降解产物的总称，FDP升高提示纤溶亢进。研究发现，COVID-19患者初始凝血功能障碍主要表现为D-二聚体和纤维蛋白原/FDP显著升高[13]。纤维蛋白原作为急性反应蛋白，在COVID-19患者发病早期即可升高，其中位水平为4.55 g/L，而在死亡患者病程晚期明显降低，有研究表明重型/危重型COVID-19患者纤维蛋白原水平的进行性下降与死亡密切相关[10],[14]。

3. 血小板：血小板的主要作用是在损伤血管内皮处形成初始凝块启动初期止血。目前研究认为，血小板也是炎症、先天性及适应性免疫的关键效应细胞，在组织损伤或感染条件下参于炎症反应，与抗原提呈细胞、淋巴细胞亚群等相互作用，并可作为免疫效应细胞介导凝血和血栓形成[15]。COVID-19患者中PLT减少占5％～36％，与重症和预后不良相关[4],[16]–[17]。有研究表明，PLT减少可作为COVID-19疾病进展及死亡的独立危险因素，入院时血小板减少患者的死亡风险是无血小板减少患者的3倍[18]–[19]。但另有研究证实COVID-19可显著改变血小板基因表达，触发强大的血小板高反应性，SARS-CoV-2感染期间出现的血小板反应性增加可导致免疫血栓（immunothrombosis）形成[20]。基于血小板在止血、炎症和免疫防御中的重要作用，目前认为PLT减少和增加都表明炎症状态增强，其中PLT减少提示血栓形成导致血小板消耗，而PLT增加可提示CSS及内皮损伤，因此动态监测PLT可能更有助于了解患者疾病严重程度及预后[21]。

4. 凝血酶原时间（PT）和活化部分凝血活酶时间（APTT）：COVID-19患者PT和APTT主要表现为正常或略延长，仅有5％和6％的COVID-19患者分别存在PT和APTT延长[2],[14],[22]。研究发现，在COVID-19危重型及死亡患者中PT明显延长，死亡患者比存活患者PT延长1.9 s，且47.6％死亡患者PT可延长6 s以上[10],[14],[23]。APTT在COVID-19危重型与非危重型患者中差异无统计学意义，其与疾病严重程度及病死率无显著相关性[10],[24]。

5. 其他凝血指标：重型/危重型COVID-19其他凝血功能指标改变有：凝血因子Ⅷ活性增强、von Willebrand因子（vWF）水平升高的同时伴有部分vWF调节蛋白酶ADAMTS13降低、抗凝血酶轻度降低以及蛋白C轻度升高等[2],[25]。另有研究发现，COVID-19的凝血功能障碍存在较一致的血栓弹性图（TEG）参数改变，即MA值增加提示凝块强度高、LY30纤溶时间缩短提示纤溶活性减低或完全停止，因此使用TEG评估COVID-19患者的高凝状态可能更助于疾病管理[26]。

二、重型/危重型COVID-19细胞因子水平改变

COVID-19患者血浆中存在多种细胞因子水平升高，且危重型患者升高的程度更明显，提示CSS可能是导致病情恶化的重要原因[3]。

1. IL-6：IL-6是重要的促炎细胞因子，可在炎症初始阶段合成，诱导纤维蛋白原、C反应蛋白、血清淀粉样蛋白A等多种急性时相反应蛋白，有助于宿主防御[27]。目前认为，IL-6是CSS的主要触发因子。有21％～52％的COVID-19患者存在IL-6水平升高，而危重型患者中比例高达74.2％[4],[28]。研究发现，IL-6可作为预测COVID-19严重程度的独立危险因素，当IL-6水平>32.1 ng/L时患者更有可能发生严重并发症[29]。另有研究表明，COVID-19危重型患者IL-6较重型患者升高3.5倍、较普通型患者升高14倍，当IL-6水平≥37.65 ng/L时可有效预测住院患者死亡[28]。

2. IL-8：IL-8又称为趋化因子CXCL-8，对中性粒细胞具有直接趋化和启动作用，是重要的炎症介质[30]。研究证实，COVID-19重型及危重型患者支气管肺泡灌洗液的髓系细胞中存在IL-8高表达[31]。IL-8可诱导中性粒细胞胞外诱捕网（NETs）释放，由中性粒细胞释放的NETs可因调控失常促进炎症加重和微血管血栓形成，研究发现其特异性标志物髓过氧化物酶-DNA（MPO-DNA）、瓜氨酸组蛋白H3（Cit-H3）水平与COVID-19的疾病严重程度相关[30],[32]。另外，IL-8还与脓毒症的发病机制以及DIC中凝血紊乱的严重程度密切相关[33]。

3. IL-10：IL-10是一种可减弱炎症反应的免疫细胞因子，也是早期抗病毒反应的关键调控因子，可决定感染是否迅速解决或进展为慢性感染[34]。研究发现，随着COVID-19疾病严重程度的加重，IL-10水平明显升高[35]。Han等[36]对武汉地区不同临床分型的COVID-19患者研究发现，危重型患者IL-10水平显著高于普通型及重型患者，且与C反应蛋白水平呈正相关，可作为COVID-19疾病严重程度的预测指标。

4. TNF-α：TNF-α是一种关键的促炎细胞因子，可介导细胞增殖、分化和凋亡等多种过程，参与各种急慢性炎症的病理反应。研究发现，COVID-19重症患者TNF-α水平较轻症患者显著升高[4],[24],[37]。COVID-19患者TNF-α水平与T细胞数量呈负相关，提示TNF-α可能直接参与诱导COVID-19患者的T细胞生成减少和衰竭[38]。另有研究发现，COVID-19中TNF-α和干扰素-γ（IFN-γ）可通过协同作用诱导炎性细胞坏死、细胞凋亡以及细胞广泛凋亡途径（PANoptosis），从而导致组织损伤和重要器官衰竭[39]。

5. IL-1β：IL-1β也是重要的促炎细胞因子之一，促进多种细胞因子（如IL-6、TNF-α等）的产生，IL-1β还可通过诱导内皮细胞中趋化因子和黏附分子的表达增加，促进免疫活性细胞向损伤组织浸润[40]。研究发现，COVID-19患者较健康人群血浆中IL-1β水平升高，且IL-1β转录物显著上调[24],[41]。但IL-1β水平可能与COVID-19的严重程度无显著相关性[42]。

6. 其他细胞因子：COVID-19患者血浆IL-7、IL-9、IFN-γ、单核细胞趋化蛋白-1（MCP-1）、粒细胞集落刺激因子（G-CSF）、粒细胞-巨噬细胞集落刺激因子（GM-CSF）、血管内皮生长因子（VEGF）等水平均高于健康人群，ICU患者血浆IL-2、IL-7、MCP-1、G-CSF等水平高于非ICU患者[24]。另外，IL-17也可作为COVID-19疾病严重程度的生物标志物[43]。

三、凝血功能障碍、CSS促进重型/危重型COVID-19患者疾病进展

重型/危重型COVID-19患者可出现严重炎症和多器官功能障碍，且进展的快速程度与炎性细胞因子及其他炎症生化标志物的急剧升高相一致，符合CSS，有学者将之称为COVID-19相关细胞因子风暴综合征（COVID-CSS），而血栓形成通路的深度激活可能是其特征之一[44]。

1. COVID-19免疫血栓形成与血栓炎症：免疫血栓形成，是指在某些情况下微血管内血栓形成具有重要的生理性免疫防御功能，具体是指单核细胞、中性粒细胞等先天性免疫细胞和组织因子（TF）等特定血栓分子介质，形成的血管内支架以识别病原体并遏制其扩散，但若调控失常可促进病理性血栓形成[45]。研究发现，CSS参与COVID-19广泛肺血管免疫血栓的形成[14]。目前认为，凝血系统和先天免疫系统相互作用，特别是单核细胞、巨噬细胞和中性粒细胞之间的相互作用是COVID-19免疫血栓形成的主要特征[46]。免疫血栓形成是先天免疫的重要部分，但血栓炎症（thromboinflammation）可促进疾病进一步发展。血栓炎症的核心机制是内皮细胞失去正常的抗血栓以及抗炎功能，导致微血管中的凝血、补体、血小板激活和白细胞募集失调[47]。研究表明，高凝性血栓炎症与CSS引起的严重炎症状态有密切关系，共同促进重型/危重型COVID-19患者的疾病进展[48]。

2. COVID-19免疫激活和凝血功能障碍：SARS-CoV-2进入机体后可直接与Ⅱ型肺泡细胞及内皮细胞上其受体血管紧张素转化酶2（ACE2）相结合，导致屏障功能障碍和通透性增加，同时触发炎症反应激活T细胞、中性粒细胞、单核-巨噬细胞和血小板活化，从而引起炎性细胞因子（IL-1、IL-6、IL-10、TNF-α等）释放、单核细胞来源的TF和纤溶酶原激活物抑制物-1（PAI-1）表达，最终形成由纤维蛋白、NETs和血小板组成的微血管和大血管血栓[49]。

研究发现，TF表达及释放在COVID-19凝血功能障碍的发展中起关键作用，单核-巨噬细胞表达的ACE2受体可直接诱导这些细胞TF过表达，炎性细胞因子和病毒特异性Toll样受体激动剂也可诱导TF过表达[50]。COVID-19凝血级联激活的主要机制是TF通路，这一过程可能是由IL-6、TNF-α及IL-1等细胞因子介导，随后导致凝血酶、纤维蛋白生成，凝血酶-抗凝血酶复合物、纤维蛋白肽增加，纤溶系统激活后可导致凝血和纤溶调控失衡[51]。

3. 凝血功能障碍与CSS共同促进COVID-19疾病进展：在既往CSS介导的人畜共患冠状病毒感染的严重急性呼吸综合征（SARS）和中东呼吸综合征（MERS）研究中发现，凝血级联失调和随后形成的肺泡内或系统性纤维蛋白凝块形成是疾病进展的重要表现[52]–[53]。COVID-CSS主要表现为IL-1β、IL-6、IL-7、IL-8、IL-9、IL-10、TNF-α、IL-17、MCP-1、GM-CSF及IFN-γ等细胞因子高水平释放，细胞因子是免疫系统的信使分子，其水平升高亦可引起巨噬细胞活化综合征（MAS）样反应，使COVID-19患者炎症反应增强，并触发内皮细胞、巨噬细胞、中性粒细胞浸润及TF表达，在凝血活化中具有重要的作用，并促进肺内凝血功能障碍和微血管血栓形成[14],[54]。

研究发现，重型/危重型COVID-19患者D-二聚体异常升高的同时可伴有IL-2R、IL-6、IL-8、TNF-α水平增高[55]–[56]。而在COVID-19 ARDS患者中发现，纤维蛋白原和IL-6水平升高之间存在明显的相关性[57]。上述临床研究结果显示COVID-19患者凝血功能障碍与CSS共同促进了COVID-19疾病进展，同时提示COVID-19患者CSS的显著差异可能与危重型患者凝血功能模式的显著差异有关。研究表明，IL-6不仅在细胞因子风暴中起核心作用，还是COVID-19凝血功能障碍的关键激活因子[14]。IL-6可以增加TF、纤维蛋白原、凝血因子Ⅷ、vWF的表达，激活内皮细胞和促进血小板的产生，以及通过降低止血抑制剂如抗凝血酶和蛋白S的水平，诱导血栓形成前状态[58]。另外，在COVID-19重症患者中的IL-6、IL-1及TNF-α与凝血激活和凝血酶生成相关，IL-1β及TNF-α可诱导PAI-1释放、减少组织型纤溶酶原激活剂（t-PA）释放，而t-PA可抑制血凝块降解[30],[59]。IL-1β还可下调血栓调节蛋白导致抗凝蛋白缺陷，特别是削弱蛋白C的激活，在减弱血栓调节蛋白抗炎活性的同时抑制抗凝作用[60]。IL-8除诱导NETs引起炎症加重及微血栓形成外，还可通过触发血小板激活来增强促凝作用[30],[33],[61]。相反，凝血途径的激活也可导致促炎细胞因子的过量产生，在肺损伤情况下，凝血酶可通过蛋白酶活化受体（PARs）增加炎症反应，诱导大量细胞因子产生[62]–[63]。这种CSS与凝血功能障碍的相互作用，促进重型/危重型COVID-19患者的炎症加重和促凝-抗凝系统失衡，形成恶性循环，最终导致DIC、多器官功能衰竭，甚至死亡[3],[30]。

四、小结

综上所述，COVID-19特征之一是与急性炎症反应相关的凝血功能障碍，患者高凝状态、微血栓形成合并CSS是疾病急速进展的关键。重型/危重型COVID-19患者凝血功能障碍与CSS相互作用，可导致患者凝血功能障碍和炎症反应持续加重，形成恶性循环，进一步加速疾病进程。目前，其具体机制还需进一步阐明。临床上应动态监测重型/危重型COVID-19患者D-二聚体、纤维蛋白原、细胞因子等指标，及时判断疾病进程及预后，必要时给予抗凝或抑制细胞因子等治疗。
